# Diseased Breath

**Published:** 1893-05

**Authors:** 


### Editorial, Etc.

DISEASED BREATH.—This subject is not alluded to in any dental text books or dental literature at command. Decaying bone in all parts of the body, when exposed to the air, exhales a fetor. The same destructive process in the teeth occasions less odor than decay in other bony tissues. When fetid breath is associated with decayed teeth, it is usual to assume that they alone are responsible; this, however, is not the case. The decaying teeth are but accessories in producing the fetor.

An offensive breath is a functional disorder liable to occur at all periods of life. It is mainly to a disarrangement of the functions of digestion and assimilation that we must look for the origin of "bad breath." In a healthy state when every organ is working naturally, there is no unpleasant odor from the expired air, but as soon as the machinery gets out of order—as soon as extraneous materials are added—the breath is tainted. We may illustrate by taking the well-known effects of various volatile substances intruded into the system. Balsam-copaiba, in small doses, passes off by the kidneys; in large doses the lungs assist in its elimination, and its presence is then readily detected in the breath. Sandal-wood oil will affect the breath in thirty minutes from the time it is swallowed. Turpentine may be noticed in less than an hour. Sulphur will produce a very marked odor in the perspiration and expired air in two hours. The characteristic and more familiar odor of alcohol is easily recognized in the breath in ten minutes. These drugs pass off through the lungs when ordinary emunctories are overworked.

In the same manner gaseous results of decaying tissue may be carried off when the other gate-ways are closed. A little sulphur in excess, combined with the watery vapor going off during expiration, forms sulphuretted hydrogen, and causes at once an offensive breath. The products of decay or "destructive assimilation" are carried off, as is well-known, by the kidneys, by the lungs, by the bowels. Some gases are manufactured in the intestinal canal by the decomposition of undigested food. These gases are secreted by the mucous membrane of the intestines; sometimes they are thrown off by the glandular apparatus in the skin. Outside the body nitrogenized substances undergo decay as they do inside. They pass through similar changes whether decomposing in the intestinal canal—the follicles of the mucous membrane—or in the cavities of the teeth. Coincident with the process of putrefaction fetid gases may be secreted by the intestines. These facts being understood it may be stated as a general proposition, that any morbid condition of the system, which prevents the passage of decomposing tissue through the bowels, will produce fetid breath. Nature to get rid of the poisonous accumulations, to maintain an equilibrium, must throw them off elsewhere, either in their offensive form or in modified, non-offensive combinations. Or, when the waste of tissue exceeds the repair, as in chronic, debilitating diseases and fevers, the eliminating glands are unable to do the work imposed upon them and vicarious elimination necessarily follows. Thus the exhalations from the skin become more or less poisoned and fetid from admixture with foul-smelling gases. The bad odor in expired air is especially noticeable, but a close examination will make known a very disagreeable odor from the skin.

The various diseased conditions which prevent the intestinal glands from eliminating the products of destructive tissue are mental emotions, constipation, indigestion, congenital deficiency in the elementary glandular system, general debility, and low forms of fevers. The local causes are decayed teeth, caries of the nasal or maxillary bones, ulceration of the lining membrane of the nose, mouth, pharynx, larynx, trachea, bronchial tubes, or "putrid bronchitis." Chronic poisoning from lead, arsenic, or mercury may also be enumerated as a common cause of diseased breath.

Bad breath arising from putrid inflammation of the mouth is a comparatively rare affection. In scorbutic ulceration of the mouth the breath is also fetid. Syphilitic inflammation and ulceration of the mouth and fauces are always accompanied by a bad breath. The odor, it is claimed, is not alone due to the secretion from the surface of the ulcer, but also to some change in the secretion of the salivary glands. R. G.

